# Optimization principles of dendritic structure

**DOI:** 10.1186/1742-4682-4-21

**Published:** 2007-06-08

**Authors:** Hermann Cuntz, Alexander Borst, Idan Segev

**Affiliations:** 1 Wolfson Institute for Biomedical Research, Department of Physiology, University College London, London, UK; 2 Department of Physiology, University College London, London, UK; 3 Max-Planck Institute of Neurobiology, Department of Systems and Computational Neurobiology, Martinsried, Germany; 4 Bernstein Center for Computational Neuroscience, Munich, Germany; 5 Interdisciplinary Center for Neural Computation, Hebrew University, Jerusalem, Israel; 6 Department of Neurobiology, Hebrew University, Jerusalem, Israel

## Abstract

**Background:**

Dendrites are the most conspicuous feature of neurons. However, the principles determining their structure are poorly understood. By employing cable theory and, for the first time, graph theory, we describe dendritic anatomy solely on the basis of optimizing synaptic efficacy with minimal resources.

**Results:**

We show that dendritic branching topology can be well described by minimizing the path length from the neuron's dendritic root to each of its synaptic inputs while constraining the total length of wiring. Tapering of diameter toward the dendrite tip – a feature of many neurons – optimizes charge transfer from all dendritic synapses to the dendritic root while housekeeping the amount of dendrite volume. As an example, we show how dendrites of fly neurons can be closely reconstructed based on these two principles alone.

## Background

The anatomy of the dendritic tree is one of the major determinants of synaptic integration [1-6] and the corresponding neural firing behaviour [7,8]. Dendrites come in various shapes and sizes which are thought to reflect their involvement in different computational tasks. However, so far no theory exists that explains how the particular structure of a given dendrite is connected to their particular function. Because dendrites are the main receptive region of neurons, one common requirement for all dendrites is that they need to connect with often wide-spread input sources such as elements which are topographically arranged in sensory maps [9]. This implies that the distance of different synaptic inputs to the output site at the dendritic root may vary dramatically from one synapse to the other. As a result, the impact of different synapses on the neural response would be expected to be highly inhomogeneous. Some neuron types seem to cope with this problem by increasing the weights of distal synapses [10-12], but see [13]. The intrinsic structure of dendrites, with thinner dendrites (larger input impedance) at more distal sites, however plays a crucial role in compensating for the charge loss from distal synapses [14-16]. In the present study we show how the effort of homogenizing synaptic efficacy can completely characterize the fine details of dendritic morphology, using the dendrites of lobula plate tangential cells of the fly visual system as an example. These interneurons integrate visual motion information over a large array of columnar elements arranged retinotopically as a spatial map [17]. By observation, their planar dendrites which spread across the lobula plate to contact the columnar input elements within their receptive fields are regarded as being anatomically invariant [18] suggesting a rather strong functional constraint.

## Results and Discussion

**Figure 1:**
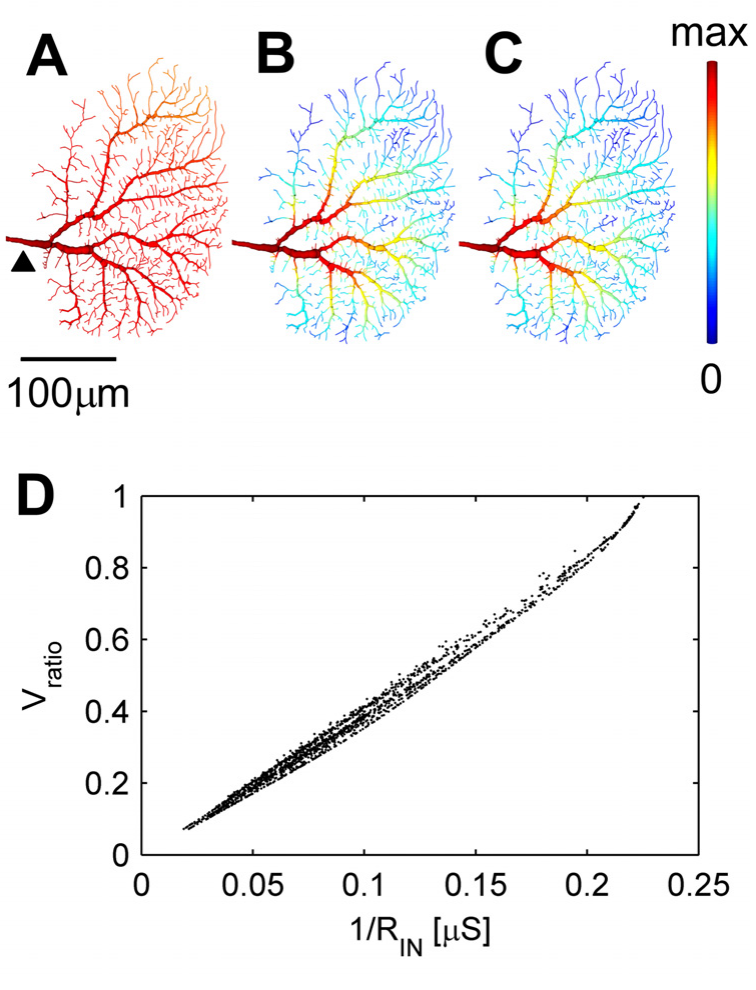
Equalization of charge transfer in a model of a reconstructed HSS cell of the fly visual system. (A) Current transfer from all dendritic locations to the dendritic root. (B) Local input conductance (inverse of input resistance, 1/*R*_*IN*_*(x)*). (C) Ratio of voltage at the dendritic root and the voltage generated at the dendrite locations, where the input current is applied. (D) Voltage ratios plotted against the inverse of the local input resistances follow a linear relationship expressing the proportionality suggested by equation (1). Colour scale in A-C ranges from 0 (blue), to maximal (red) current transfer (A), input conductance (B) and voltage ratio (C). Reference point for dendritic root is indicated by an arrow in A.

Using detailed morphologically and physiologically realistic compartmental models of tangential cells [19,20] we calculated the passive steady state current transfer between all dendritic locations and the root. We found that the current transfer from all dendritic locations to the axonal summation point is strongly equalized throughout the dendrite (Figure 1A). This corresponds well with findings on many other cell types, notably CA3 pyramidal neurons and Purkinje cells [14,16]. In principle, the root voltage response (*V*_*root*_) to a constant steady synaptic current (*I*_*syn*_) at each synapse location, *x*, would become independent of that synaptic site if the ratio of the voltages between the dendritic root and the location *x V*_*ratio*_*(x) *(also called attenuation factor [2]) was reciprocally related to the input resistance (*R*_*IN *_*(x)*) at the synapse location *x*:

*V*_*root*_(*x*) = *V*_*ratio*_(*x*)·*R*_*IN*_(*x*)·*I*_*syn *_

We therefore investigated whether such an inverse proportionality between the voltage ratio and the local input resistance exists. As can be seen from Figure 1B–D for tangential cell dendrites, the input resistance does indeed increase in a similar way to the voltage ratio drop off throughout the dendrite. The inverse proportionality between *R*_*IN *_and *V*_*ratio *_is reflected in their relationship to each other (Figure 1D). This observation holds true when strong full-field visual stimulation increases the membrane conductance drastically (see Additional file 1, Figure S2) and when peak or integral values of the charge are considered for time varying synaptic currents. This feature of the passive dendritic structure represents a homogenous backbone on which active properties could sensibly implement non-linear computations. However, in the case of the cells analysed here, responses correspond to graded potential shifts only moderately further modulated by active non-linearities. In the following we will explain this behaviour of the passive dendritic tree by first considering the effect of diameter tapering and then examining the topological features.

### Diameter tapering related electrotonic homeostasis

The increasing input resistance in distal dendrites producing an almost homogenous current transfer could be a simple consequence of the decrease in dendrite diameter with distance from the root [2]. In a symmetrical dendritic tree corresponding to a cylinder of constant diameter, the increase of *R*_*IN *_with distance, as well as the attenuation factor can be computed analytically [2]. There, *R*_*IN *_and the attenuation factor are not inversely proportional since their ratio depends on *cosh(L)*, *L *being the electrotonic length (in units of the space constant, λ). This implies that tangential cells and other neurons which optimize current transfer from synapses to dendritic root achieve this by utilizing different principles.

**Figure 2:**
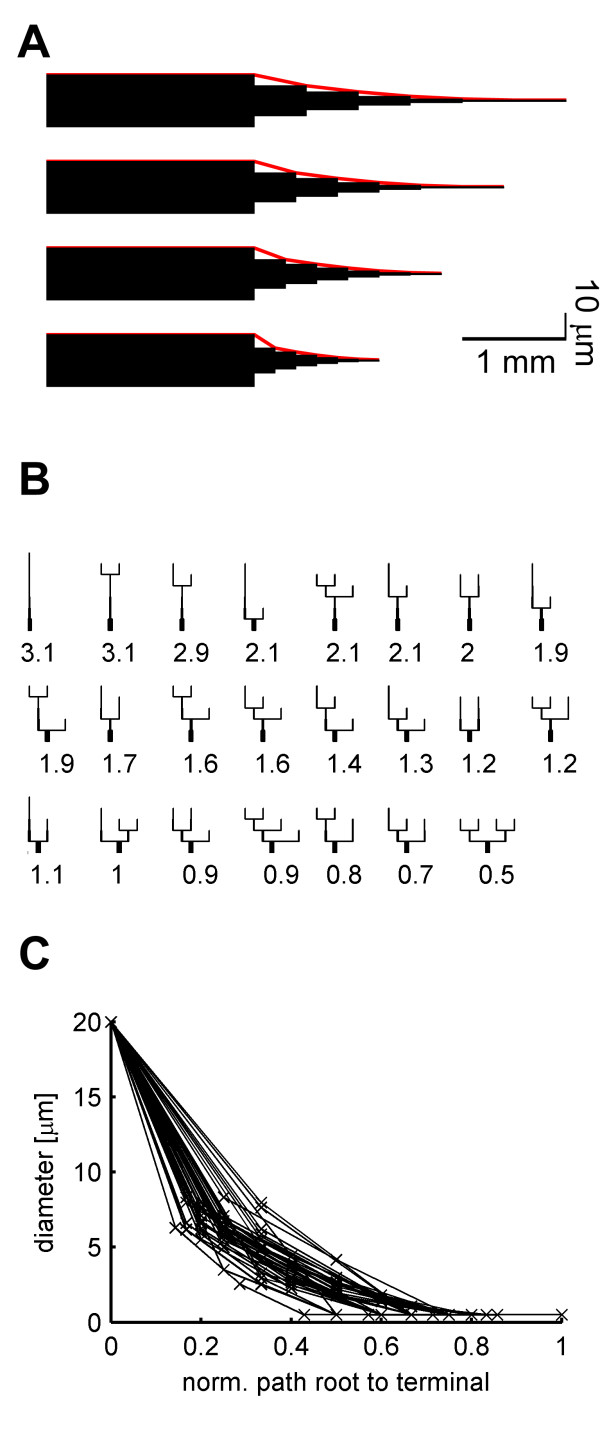
Diameter optimization for optimal current transfer in simplified dendritic cable models. (A) Optimal diameters for maximal charge transfer in four unbranched cable models, each composed of six segments of equal length (200, 300, 400 and 500 μm from bottom to top) and all attached to a cylindrical axon (20 μm in diameter, 2 mm long). Scale x : 1 mm y : 10 μm. Red: diameter tapering. (B) Diameter optimization in branched structures with six 300 μm-long segments each, sorted by error size (marked values) as defined in Equation (4). Part of the axon at the bottom of each tree is cut for presentation. (C) Dendrite diameter tapering for all models shown in B, when the path from root to terminal is normalized.

In order to come up with optimality criteria for a location independent current transfer, we adjusted diameters in simple dendritic cable models. The models were built from six segments of equal length preceded by a 2 mm long cylinder of a fixed (20 μm) diameter representing the axon and its associated leak conductance which, in tangential cells, is directly connected to the root of the dendrites. The diameter of the individual compartments was limited to a lower bound of 0.5 μm. In unbranched cable models, optimal current transfer was obtained when the cables tapered monotonically from root to distal tip, ending in all cases with the preset lower bound (see Figure 2A). The axonal cylinder prevented "sealed end" artefacts on the proximal side. With a short axonal cylinder, the optimal initial diameter was larger than the fixed axon diameter, before decaying monotonically to the minimum at the distal site (see Additional file 1, Figure S3, for complete analysis). Similarly, in all possible branched structures composed of six segments of equal length (see Figure 2B) the current transfer was optimal with monotonically decaying diameters. The tree with the most branching (lower right) exhibited the best current transfer; this was, at least partly, due to the shorter average electrotonic distance of this tree. Interestingly, the optimization procedure assigned lower bound diameter to early termination branches as well as to distal ones, independently of the branch order. This corresponds well to observations in real cells (compare with terminal branches close to the dendritic root in Figure 1). Figure 2C summarizes the distribution of diameters in the models shown in Figure 2B, demonstrating the tendency of the diameter to decay along normalized paths from the dendritic root to all terminals. A comparison of the current transfer in models with optimized diameters and in corresponding models with constant diameters is shown in Figure S3 (Additional file 1).

**Figure 3:**
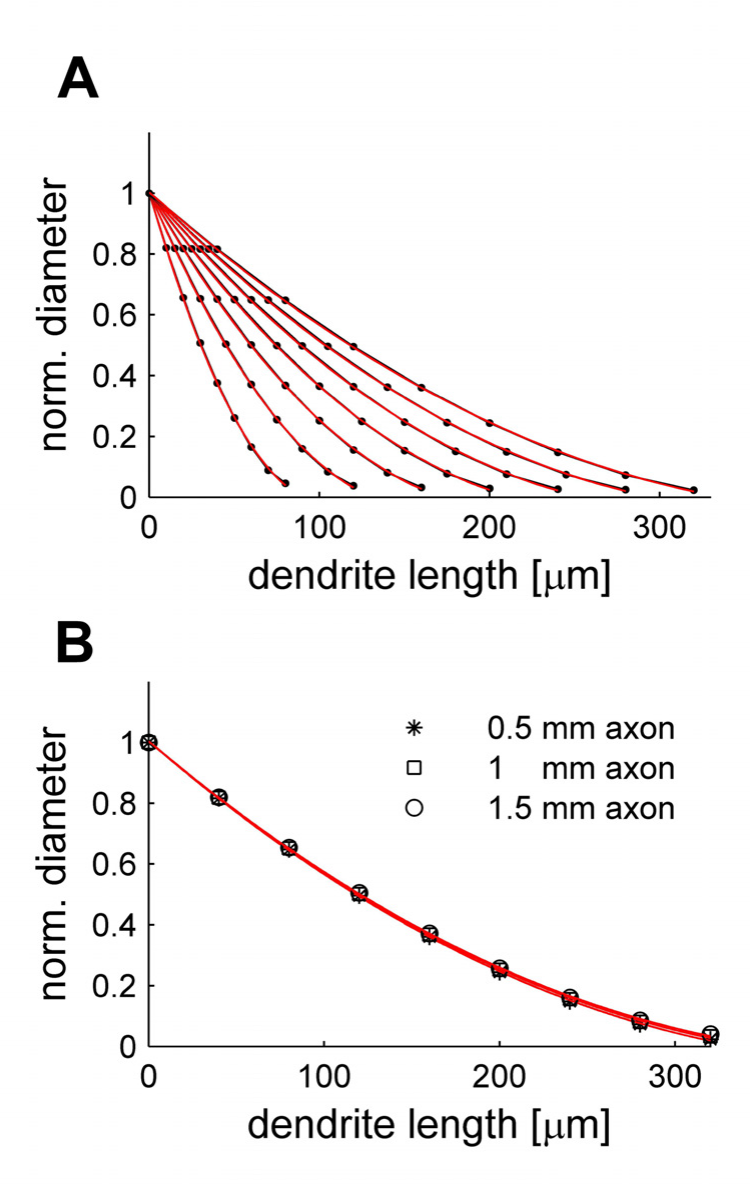
Optimal tapering follows quadratic decay. (A) Normalized optimal diameters (black dots) in cable pieces of different lengths divided into 10 segments each. In all cases a quadratic equation (red lines) could well describe the course of tapering. The fixed diameter of the first segment corresponding to the axon piece is not shown. (B) Changing the size of the leak (length of the first segment) did not alter the relative course of tapering.

In order to better observe the exact course of tapering we optimized the diameter in cable pieces of 10 segments under various parameter settings. The optimal tapering of the diameter could best be characterized by a quadratic fit in all cases. This is illustrated for varying the length of the individual segments in Figure 3A. Varying the axonal leak by changing its length did not change the relative tapering (Figure 3B); only the overall scaling of the diameters was affected.

### Synapse-targeted topological properties of dendrites

Aside from adjusting dendritic diameters to optimize synaptic efficacy, dendrites could also follow some optimization principles with respect to their branching structure. To describe the topology of dendrites, graph theory provides an appropriate framework. In this context, the branching structure of a dendrite is regarded as a network connecting all points at which synapses are located. After assigning vertices to particular locations in space according to putative synapse positions, the branching structure is defined as the set of directed edges between these locations leading away from the dendritic root. From a purely topological point of view, maximal proximity of each synapse to the dendritic root is achieved by a direct connection in a fan-like manner. This would minimize the path lengths with respect to individual synapses since each indirect connection would correspond to a detour on the way from the synapse to the dendritic root. However, such a fan-like structure is not usually observed in real dendrites.

**Figure 4:**
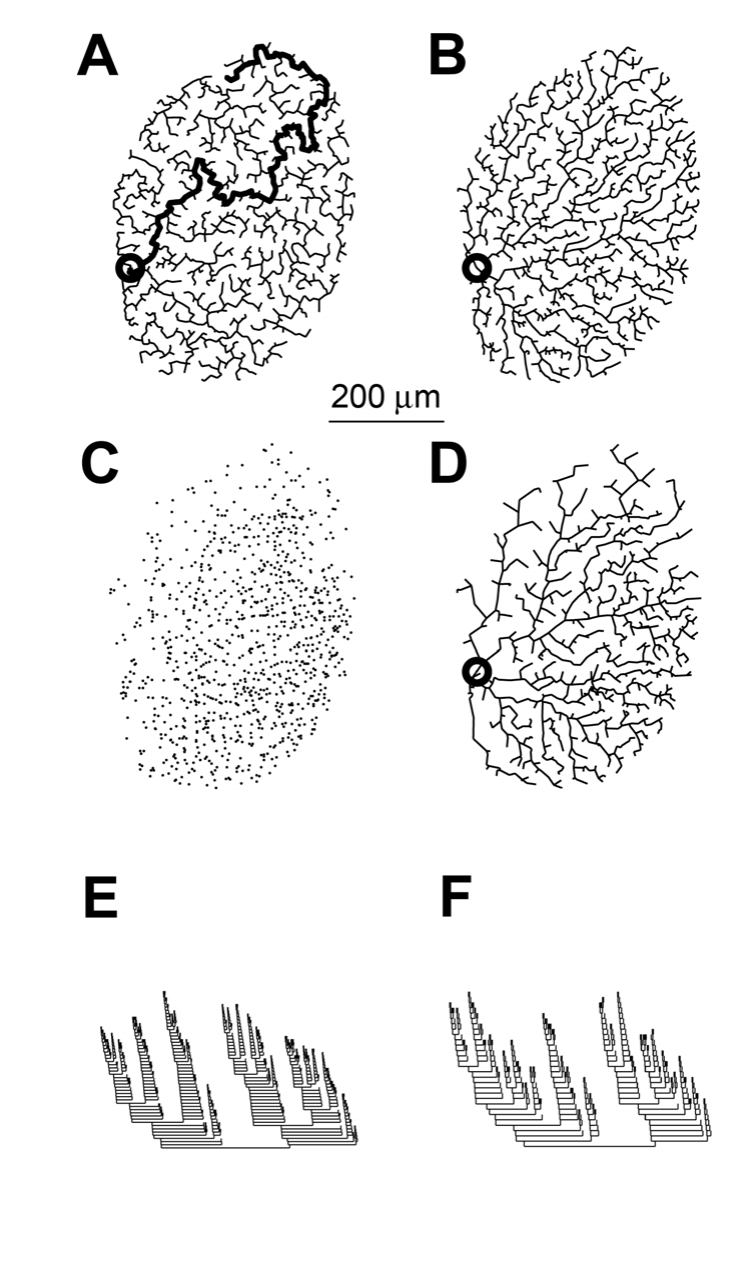
Rules for optimizing dendritic branching. (A) Minimum spanning tree for randomly distributed points in the convex hull of dendritic territory of HSS neurons. Longest path is drawn in bold. (B) Extended minimum spanning tree, minimizing both the total path length from all synaptic locations to the root and the total wiring length. (C) Branching and termination points as putative sites for synaptic contacts for the HSS dendritic tree shown in Figure 1D, same algorithm as in B, using the putative synaptic sites from C. Dendritic root is marked with a circle. (E, F) Dendrograms representing the topology of the reconstructed HSS neuron and the artificially constructed dendritic tree shown in D, respectively.

An alternative to the maximal proximity criterion is that the dendritic trees connect synaptic inputs to the dendritic root using the minimal total length of wiring [21,22]. To investigate this possibility, we used the minimum spanning tree algorithm [23], a common tool in graph theory. We first distributed random points (~2000) within the territory of the dendrites of the tangential cell from Figure 1. However, minimizing the wiring in order to connect these points proved to be an insufficient optimization constraint to reproduce structures similar to tangential cell dendrites: some points were connected in a rather long path to the dendritic root (Figure 4A). Only a combination of both optimizing the synaptic proximity to the dendritic root and minimizing the total amount of wiring lead to reasonable dendrite-like structures (Figure 4B, full analysis of branching in Additional file 1, Figures S4 and S5). To further validate this simple dendrite construction method visually, we applied the algorithm combining both wiring rules to all branching and termination points of the existing tangential cell model (Figure 4C) assuming them to be representatives for putative synapse locations (the growth progress of the arborisation in the algorithm can be seen in Additional file 2, Movie S1). The resulting structure was similar to the corresponding real cell (compare morphology in Figure 4D with that of Figure 1), as were the characteristic fractal structure of the topology (compare dendrograms in Figure 4EF). However, because the algorithm was restricted to fewer points (only branching and termination points) than the number of possible synaptic sites in the modelled cell, the reconstruction was bound to a lower spatial resolution than the original neuron. Indeed, a more complete understanding of the correct connectivity graph can only be obtained when the exact locations of synapses are known.

**Figure 5:**
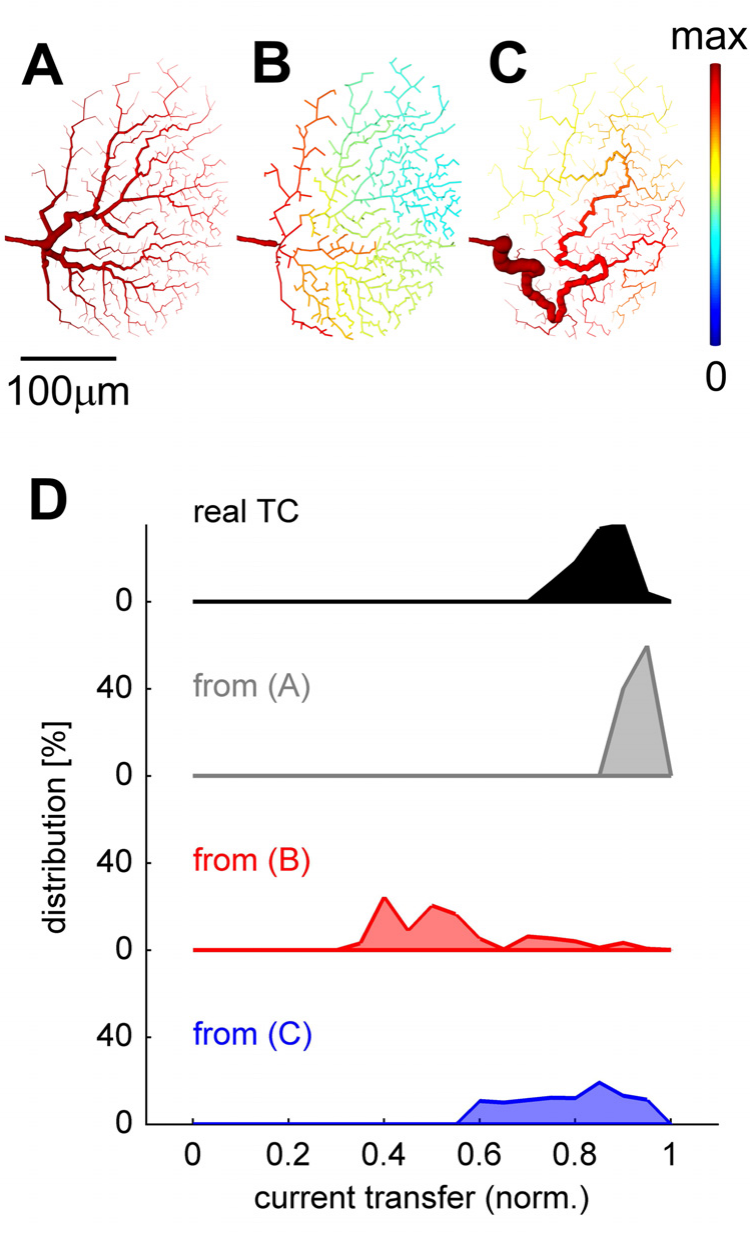
Validation and quantification for optimizing parameters. (A) Current transfer to the root in artificially constructed dendritic tree shown in Figure 4D with tapering diameters. (B) Current transfer in reconstructed HSS dendritic tree assuming constant (2.3 μm) diameter for all dendritic branches (the maximal electrotonic distance in this case was similar to that of the HSS model with tapering dendritic diameter). (C) Current transfer in reconstructed dendrite, in which dendritic topology only minimized the total wiring from root to all points shown in Figure 4C but with tapering dendritic tree. (compare these graphs with Figure 1A, all having the same colour scale). In all cases the axon of the HSS model was appended to the artificially reconstructed dendrites. (D) Distributions of normalized current transfer in the different nodes of the dendrites of the real HSS model (black) and the three reconstructed dendrites (A – grey, B – red, C – blue).

Next, we incorporated monotonically decaying diameters into the branching structures obtained with the extended minimum spanning tree algorithm. The course of tapering was set to correspond to the quadratic equations from the electrotonic optimization in single cables (as in Figure 3A). The resulting dendrites exhibited an equalized current transfer distribution similar to the one obtained from real cells (Figure 5A). If, in contrast, the diameter was kept constant throughout the dendrites the current transfer broke down rapidly (Figure 5B). Also, when topological optimization constrained only the total amount of wiring, without further minimizing the length from each synaptic site to the root, then charge transfer was not equalized (Figure 5C), implying that constraining the path length to the root is important for synaptic integration. In Figure 5D the distributions of current transfer values for all three cases are compared to the one in the real tangential cell model.

## Conclusion

Lobula plate tangential cells exhibit a rather invariant anatomy from one animal to the next [18]. They are interneurons whose function it is to integrate over an array of local columnar elements distributed retinotopically over the surface of their receptive fields. Here we propose that optimizing synaptic efficacy at the root leads to the stereotyped nature of their dendritic structures. We show that dendritic diameter tapering towards the terminal tips optimally equalizes current transfer from all synaptic locations to the dendritic root. This could correspond to the finding that dendritic morphology can be described in a diameter dependent manner [24]. The optimal course of tapering is a quadratic decay. It will be interesting to further investigate this electrotonic feature of dendrites and cables in general. In addition to its optimized diameter tapering, the dendritic tree is optimally branched to keep synaptic contacts close to the dendritic root whilst minimizing the total dendritic wiring. Our analysis has therefore re-affirmed the importance of wiring cost to which several morphological and organizational principals in the brain were attributed previously [21,25]. Together, these represent fundamental principles for shaping dendrite structure. Both monotonic tapering diameters and homogenous integration of spatially distributed inputs are characteristic of many dendrites; these principles may well therefore be applicable for many other systems. In recent years, a number of approaches have been taken to describe dendritic morphologies based on local branching statistics and on only a few branching rules [26,27]. In contrast to these studies, we show here the possibility of setting neuroanatomical reconstruction into the context of their function: synaptic integration.

## Methods

### Electrotonic investigation on dendrites as graphs

When dendrites are regarded as graphs their branching structure can be well described with the corresponding directed adjacency matrix *A*, a quadratic matrix of size *NxN *where *N *is the number of nodes in the dendritic tree (see Additional file 1, Figure S1A). In this graph the direction of the edges show away from the first node, the dendritic root, representing the arbitrary starting vertex (1). The electrical circuit containing all conductances of the dendritic tree in the matrix *G *can be written as

$$G=G_{m}\pi dl+G_{a}\left[sum\left(A\frac{\pi d^{2}}{4l}+\frac{\pi d^{2}}{4l}A^{T}\right)-\left(A\frac{\pi d^{2}}{4l}+\frac{\pi d^{2}}{4l}A^{T}\right)\right]$$

where only the axial conductances relate to the adjacency matrix *A*. *G*_*m *_and *G*_*a *_are the specific membrane and axial conductance values, respectively. *d *and *l *correspond to matrices in which the diameter and length values of individual compartments are located along the diagonal. The *sum () *term represents a matrix in which the elements of the sums over the columns are written along the diagonal. The term in the square brackets has the structure of a weighted admittance matrix. The steady-state electrotonic character of the dendritic tree can be described in the inverse of this matrix *G *[28]:

V=1G=G−1
 MathType@MTEF@5@5@+=feaafiart1ev1aaatCvAUfKttLearuWrP9MDH5MBPbIqV92AaeXatLxBI9gBaebbnrfifHhDYfgasaacH8akY=wiFfYdH8Gipec8Eeeu0xXdbba9frFj0=OqFfea0dXdd9vqai=hGuQ8kuc9pgc9s8qqaq=dirpe0xb9q8qiLsFr0=vr0=vr0dc8meaabaqaciaacaGaaeqabaqabeGadaaakeaacqWGwbGvcqGH9aqpdaWcaaqaaiabigdaXaqaaiabdEeahbaacqGH9aqpcqWGhbWrdaahaaWcbeqaaiabgkHiTiabigdaXaaaaaa@3525@

when the current matrix *I *is chosen to be the identity matrix. The resulting symmetric matrix *V *corresponds to the potential distributions throughout all nodes in each column when current is injected in the node corresponding to the column index. The local input resistances in the different branches of the dendritic tree can therefore be read in the diagonal of *V*. In order to obtain the electrotonic measurements in the tangential cell model used in Figure 1, we converted the neuroanatomical description of a compartmental model of an HSS cell [19] into a sparse adjacency matrix and sparse matrices containing length and diameter of each compartment in the diagonal. The inverse of the matrix *G *obtained from Equation (2) is shown for this compartmental model (consisting of 2251 compartments) in Figure S1B (Additional file 1). The specific passive properties (membrane resistance of 2000 Ωcm^2 ^and axial resistance of 40 Ωcm constant in all models) were adopted from [19].

### Electrotonic optimization

Reduced dendritic models with six segments were obtained from all possible non-equivalent adjacency matrices (only allowing binary branching). An axon was represented by a 2 mm long passive cylinder with a diameter of 20 μm which was appended at the dendritic root. Diameters of the other segments were optimized by minimizing current transfer along the model dendrite. This was done by injecting a current to the axon (at the root, segment #1) and measuring the potential *V*_*i *_in all segments; noting that in passive dendrites, current transfer is reciprocal with respect to injection and recording sites [29]. The error function

E=∑i=1N|1−ViV1|
 MathType@MTEF@5@5@+=feaafiart1ev1aaatCvAUfKttLearuWrP9MDH5MBPbIqV92AaeXatLxBI9gBaebbnrfifHhDYfgasaacH8akY=wiFfYdH8Gipec8Eeeu0xXdbba9frFj0=OqFfea0dXdd9vqai=hGuQ8kuc9pgc9s8qqaq=dirpe0xb9q8qiLsFr0=vr0=vr0dc8meaabaqaciaacaGaaeqabaqabeGadaaakeaacqWGfbqrcqGH9aqpdaaeWbqaamaaemaabaGaeGymaeJaeyOeI0YaaSaaaeaacqWGwbGvdaWgaaWcbaGaemyAaKgabeaaaOqaaiabdAfawnaaBaaaleaacqaIXaqmaeqaaaaaaOGaay5bSlaawIa7aaWcbaGaemyAaKMaeyypa0JaeGymaedabaGaemOta4eaniabggHiLdaaaa@3FAD@

(*N *= 7, number of segments including the appended axon) was minimized using the built-in MATLAB function *fminsearch*. Results were supported by corresponding simulations in NEURON [30]. Since segments of up to 500 μm are not isopotential, the adjacency matrix required a stretching extension to divide the seven segments into several compartments. A complete investigation of the current transfer optimization in the example of the unbranched cable showed similar results under a variety of simulation settings (Additional file 1, Figure S2). In all cases the diameter tapered in a quadratic manner starting at different initial diameters depending on the settings of the bounding axonal segment.

### Topological measures

With continuous matrix multiplication on the directed adjacency matrix, as in *A*^*r*^, the (*i*, *j*)-entry represents the number of distinct *r*-walks from node *i *to node *j *in the graph. Therefore, some elementary statistical properties, e. g. path lengths, can readily be accessed using the graph representation of the dendritic tree. To be able to compare topologies between different dendrites and assign them to an equivalence class we developed an ordering scheme based on conventional graph sorting. After assigning a root index, the remaining indices were first sorted by path length to the root and if those were the same then by level order (summing up the path lengths to the root of all child branches). Indices were then sequentially reassigned just next to their parent index following the sequence of the above order. This resulted in dendrograms in which the 'heavier' sub-tree is always on the left.

### Optimizing topological features

The extended minimum spanning tree algorithm to obtain the adjacency matrix in an optimized wiring scheme for a given set of points followed the principles described by Prim [23]. Starting with the root, the set of connected points was compared to the set of non-connected points. One at a time, the closest point from the non-connected set (the distance measure included the total path length to the root with a balancing factor *bf*) was connected to its partner in the set of connected points. In order to keep the total path length of each new point *P*_*x *_to the root *P*_0 _small, we simply added a term to the distance measure *D *weighted by a factor *bf*:

*D*_*x,i *_= |*P*_*i*_*P*_*x*_|+ *bf*|*P*_0 _→ *P*_*x*_|

*bf *was chosen to be 0.2 to reproduce best the topology of the tangential cell dendrite (for the choice of *bf *see Additional file 1, Figures S4 and S5). This represents a crude definition of the distance constraint and can be refined in further studies. The algorithm was run on homogenously distributed points in a random way confined to the convex hull around the dendrite of the original tangential cell (Figures 4AB, and Additional file 1, Figures S4 and S5). Alternatively, the branching and termination points of the original tangential cell were chosen (Figures 4CD, 5 and Additional file 1, Figure S6).

In order to apply diameter tapering on the constructed topology for Figure 5, the diameters corresponding to the optimized quadratic tapering along all normalized paths from root to terminal points were averaged for each compartment. In this way a monotonic tapering could be attributed to any type of branching structure. Validation of this procedure and comparison to the monotonic tapering in real tangential cells is shown in additional file 1, Figure S6. All computations were performed in MATLAB.

## Abbreviations

*V*_*root*_, voltage response at dendrite root; *I*_*syn*_, constant steady synaptic current; *V*_*ratio*_, attenuation factor; *R*_*IN*_, input resistance; *A*, directed adjacency matrix.

## Competing interests

The author(s) declare that they have no competing interests.

## Supplementary Material

Additional file 1 Supplemental material. Supplementary Figures S1-S6 and figure captions. Click here for file

Additional file 2 **Movie S1. **Movie illustrating the algorithm for the assembly of dendrite topology. Points from the unconnected set (black dots) are sequentially added to the existing tree, minimizing both total wiring and path to the root (black circle) along the tree structure. Click here for file
