# Supplementary material for: Optimization principles of dendritic structure
Source: Theor Biol Med Model. 2007 Jun 8;4:21. doi: 10.1186/1742-4682-4-21 (PMC1924501; doi:10.1186/1742-4682-4-21)
Supplement: Additional file 1 — Supplemental material. Supplementary Figures S1-S6 and figure captions. [file 1742-4682-4-21-S1.doc]

**Figure S1.** Calculating with dendrites as graphs. (A) Adjacency matrix representation of a dendrite topology. Dendrite topology can be represented as a set of *N* nodes connected by *N-1* edges forming a graph (left). The corresponding directed adjacency matrix is an *NxN* sparse matrix, where edges are marked by ‘1’ at the indices of the nodes they connect to (row: arrowtail, column: arrowhead). (B) Electrotonic connectivity map of the tangential cell model used for Figure 1. Symmetrical matrix *V* is obtained from Equation (3). Each row (or column) represents the potential distribution when current is injected into the node corresponding to the row (or column) index. The diagonal therefore corresponds to the local input resistance (resulting potential at a node when a current is injected there). The potential distributions in the rows and columns correspond to the current transfer.

**Figure S2.** Normalized current transfer in real TC dendrites. In the upper panel: distribution of current transfer throughout the branches of the TC dendrite as in Figure 1A of main article (black shaded area with straight line). The distribution shifts only slightly when the membrane conductance is doubled corresponding to strong whole field visual motion input (black shaded area with dotted line). In comparison in the lower panel: distribution of current transfer values throughout the dendrite when the diameter is kept constant at the mean dendrite diameter of the real TC (red shaded area with straight line). The distribution is much broader and reaches much lower values. Doubling the membrane conductance shifts this distribution in a similar way (red shaded area with dotted line).

**Figure S3.** Validation of current transfer optimization. Comparison between optimal (quadratic) diameter tapering (black) and constant diameters of 20 (red), 10 (green), 5 (blue) and 2 (grey) μm at different configurations of the axonal segment. (A) axon 2 mm reference point at 40 % axon length; axon diameter 20 μm. (B) axon 2 mm reference point at 80 % axon length; axon diameter 20 μm. (C) axon 1 mm reference point at 80 % axon length; axon diameter 20 μm. (D) axon 1mm reference point at 80 % length; axon diameter 10 μm. Black circles indicate dendritic root reference points, black brackets indicate dendrite area with malleable diameters.

**Figure S4.** Topological calibration and validation of minimum spanning tree I. Branching structure plot, dendrograms and distributions for branching order, branch length and path lengths to the root value for all branches are displayed for the different constructed minimum spanning trees as a function of their balancing factor (*bf*, between minimum wiring and minimum path lengths). These are compared to the real tangential cell in red in the first row. Best match is for *bf* between 0.1 and 02.

**Figure S5.** Topological calibration and validation of minimum spanning tree II. (A) Scholl analysis of topology. Circles are drawn around the root of the dendrite with increasing diameters. For each circle the number of intersections with the dendrite is counted. Curves are shown for minimum spanning trees on random points with balancing factor (between minimum wiring and minimum path lengths) *bf* = 0.2 and *bf*  = 0.8 and compared with the real tangential cell in red. (B) The average proportion between Euclidean direct distance and path length from the dendrite root to all branches are drawn for the different reconstructed trees as a function of *bf*. As a comparison the value for the real tangential cell is drawn in as a red dotted line at 0.73.

**Figure S6.** Comparison of artificial dendrites with real tangential cell. (A) Diameter tapering in the reconstructed tangential cell and (B) in the same model cell after assigning tapering. Black line is the average diameter tapering and gray patch outlines 1 standard deviation from the mean. (C) Diameter values in corresponding compartments in A and in B plotted against each other. Black line is the binned average (tangential cell compartmental model diameters are discrete values from 1 to 12) with standard deviation. The red line indicates the one to one relationship.

**Movie S1.** Movie illustrating the algorithm for the assembly of dendrite topology. Points from the unconnected set (black dots) are sequentially added to the existing tree, minimizing both total wiring and path to the root (black circle) along the tree structure.


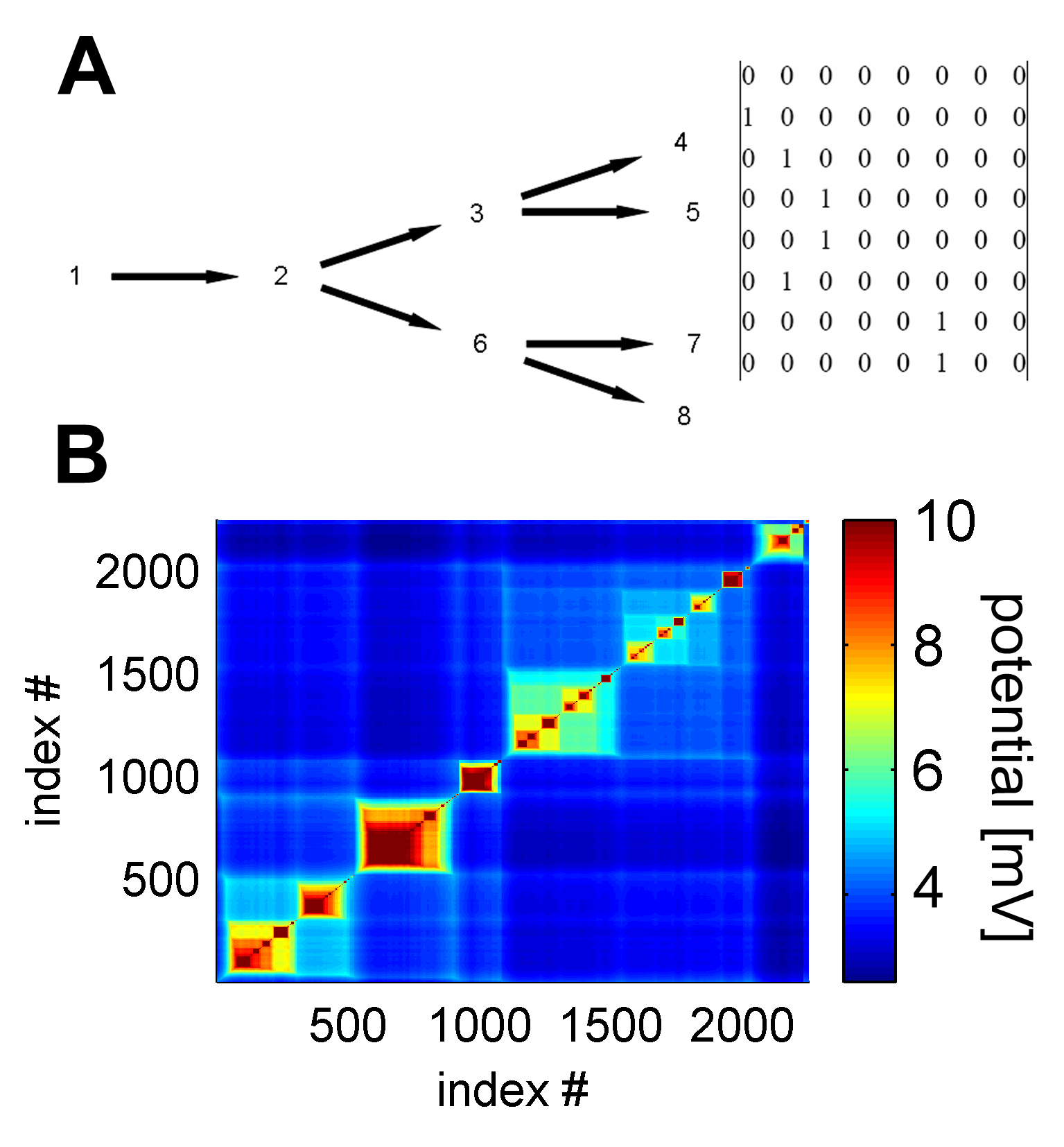


Figure S1


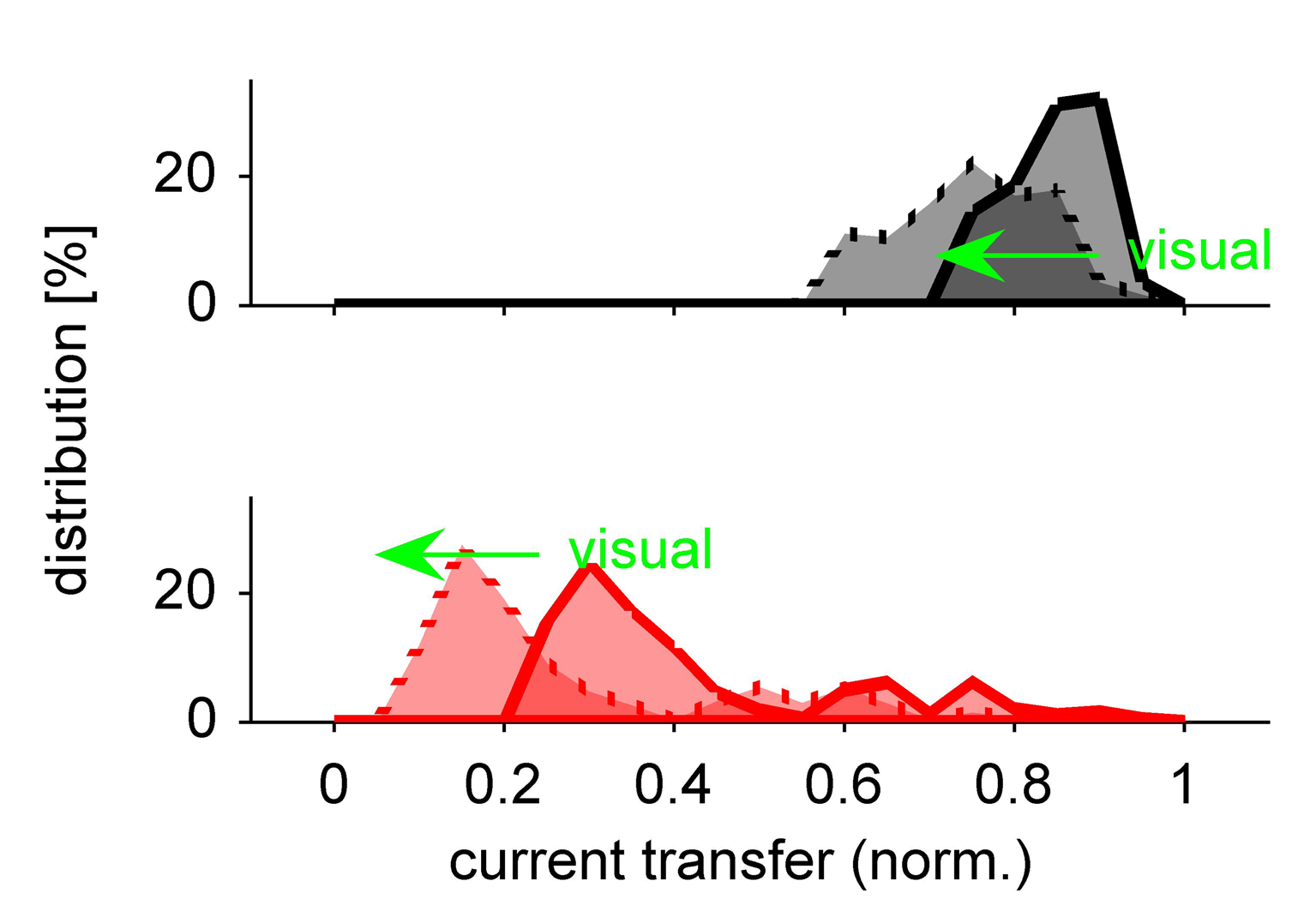


Figure S2


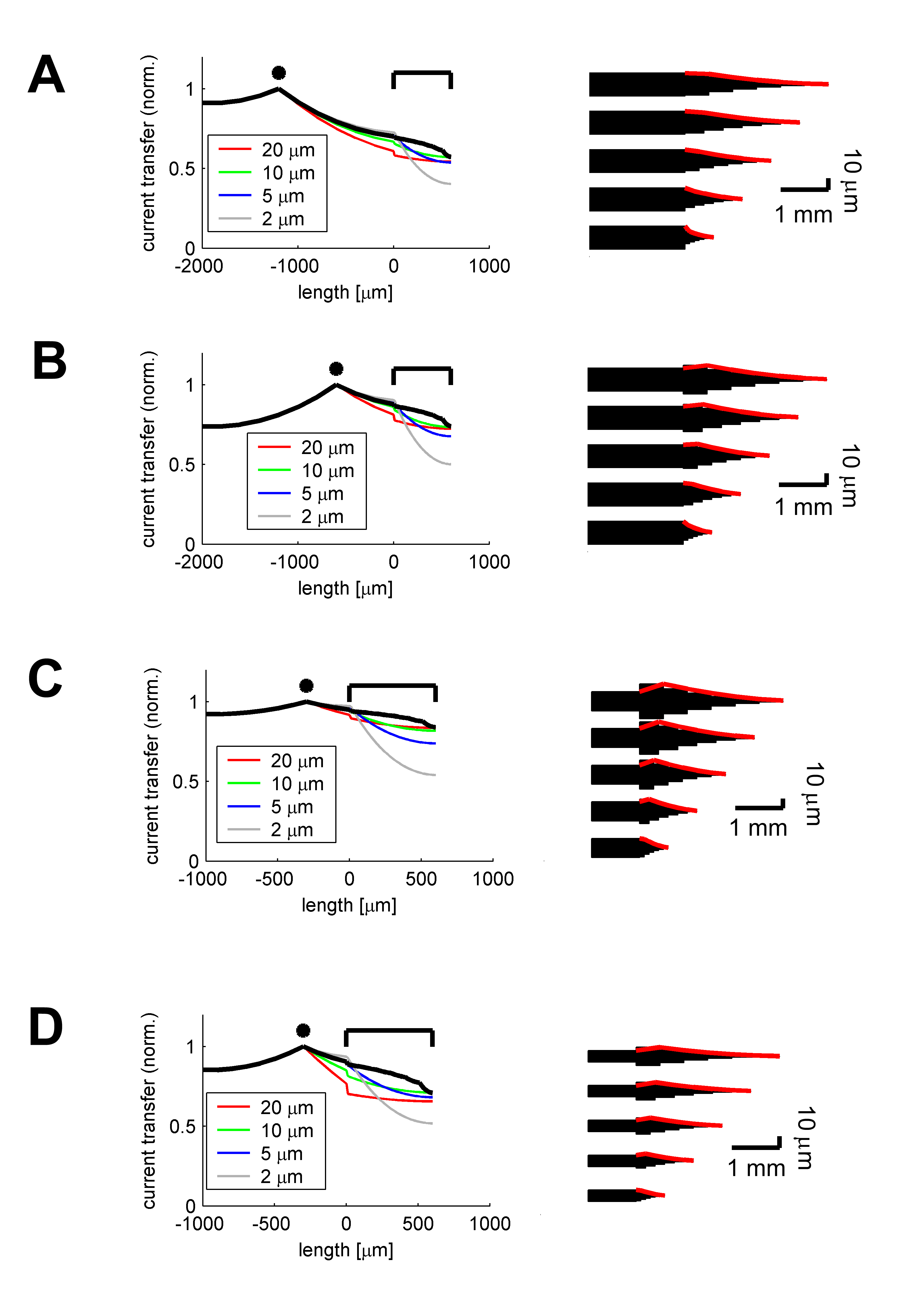


Figure S3


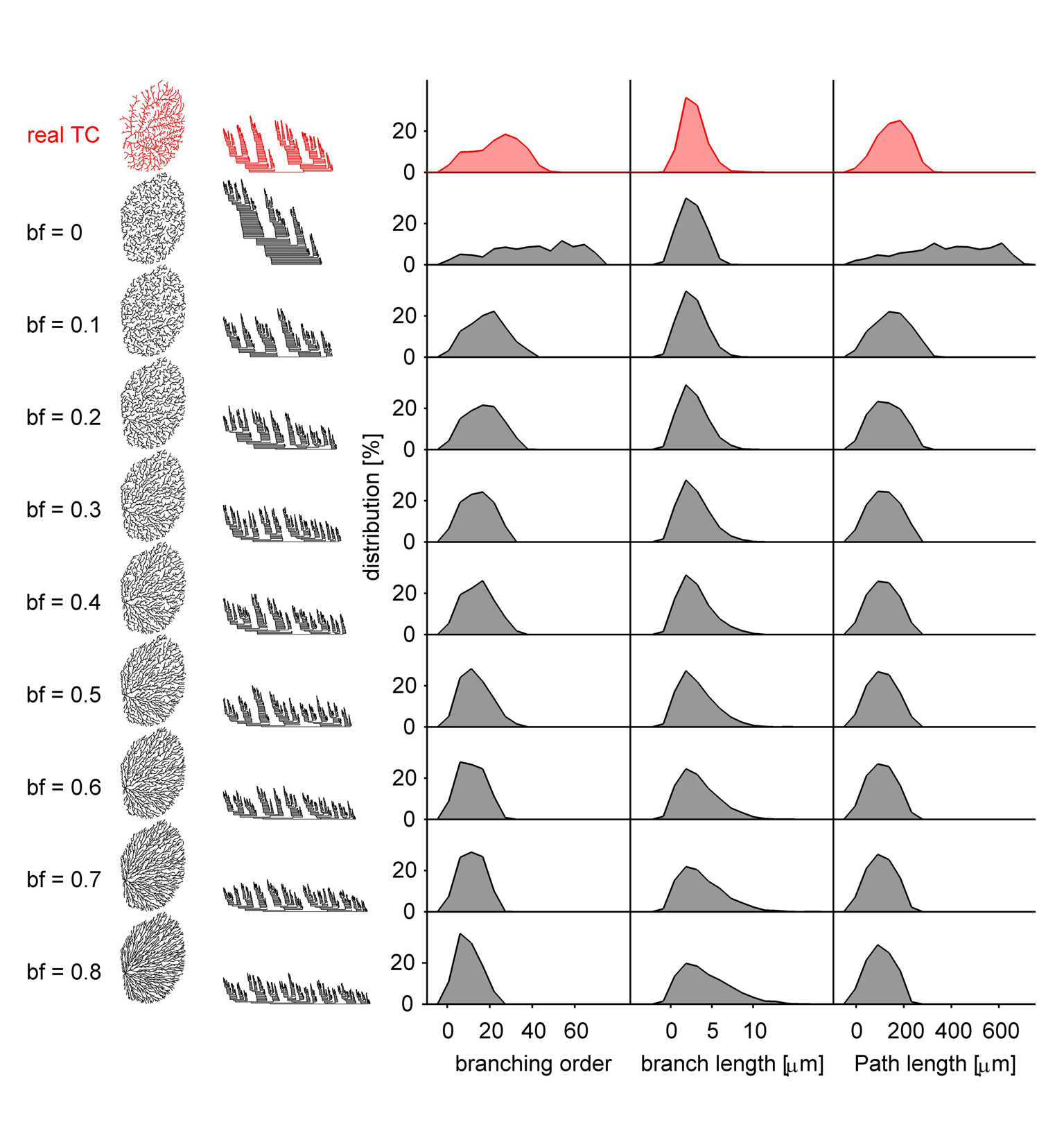


Figure S4


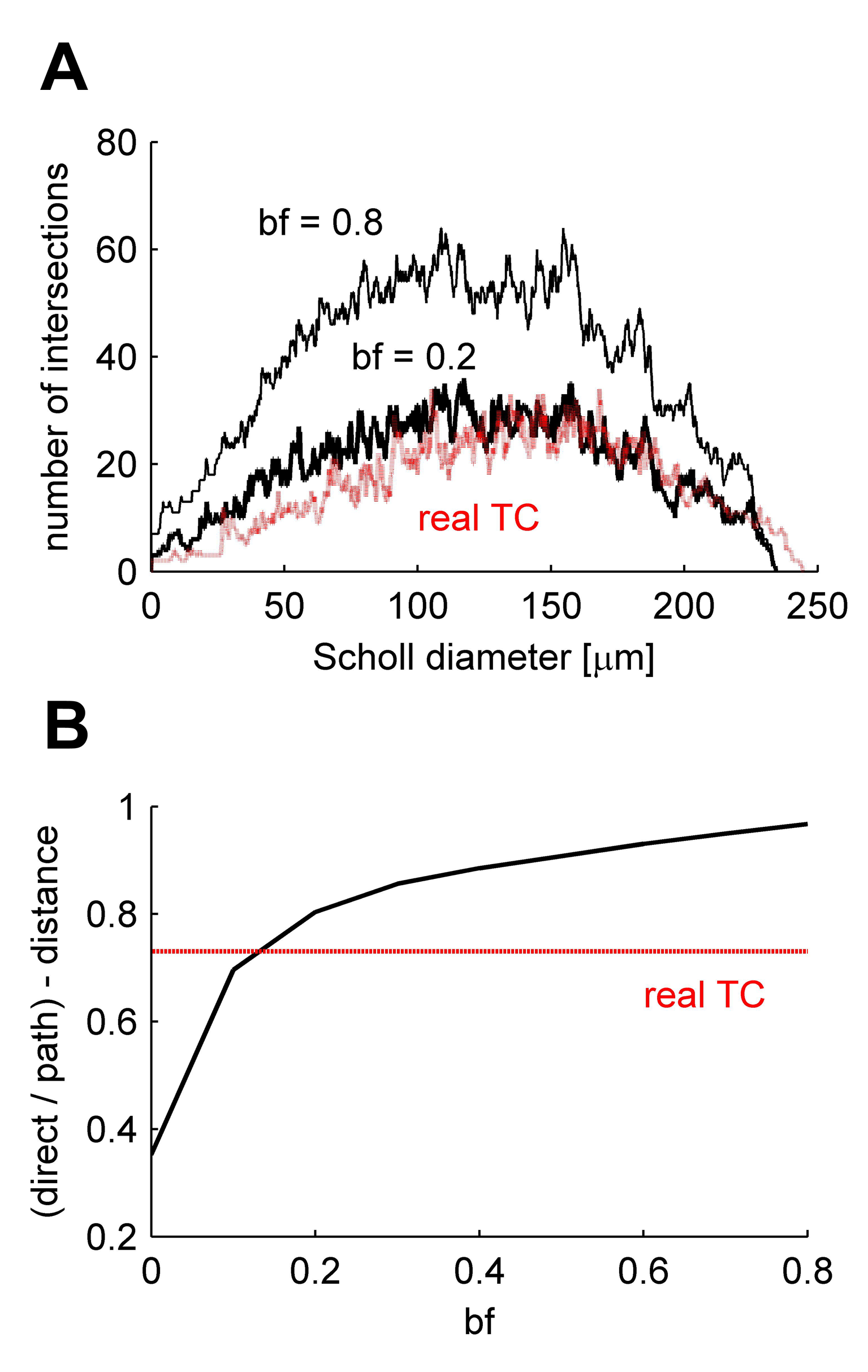


Figure S5


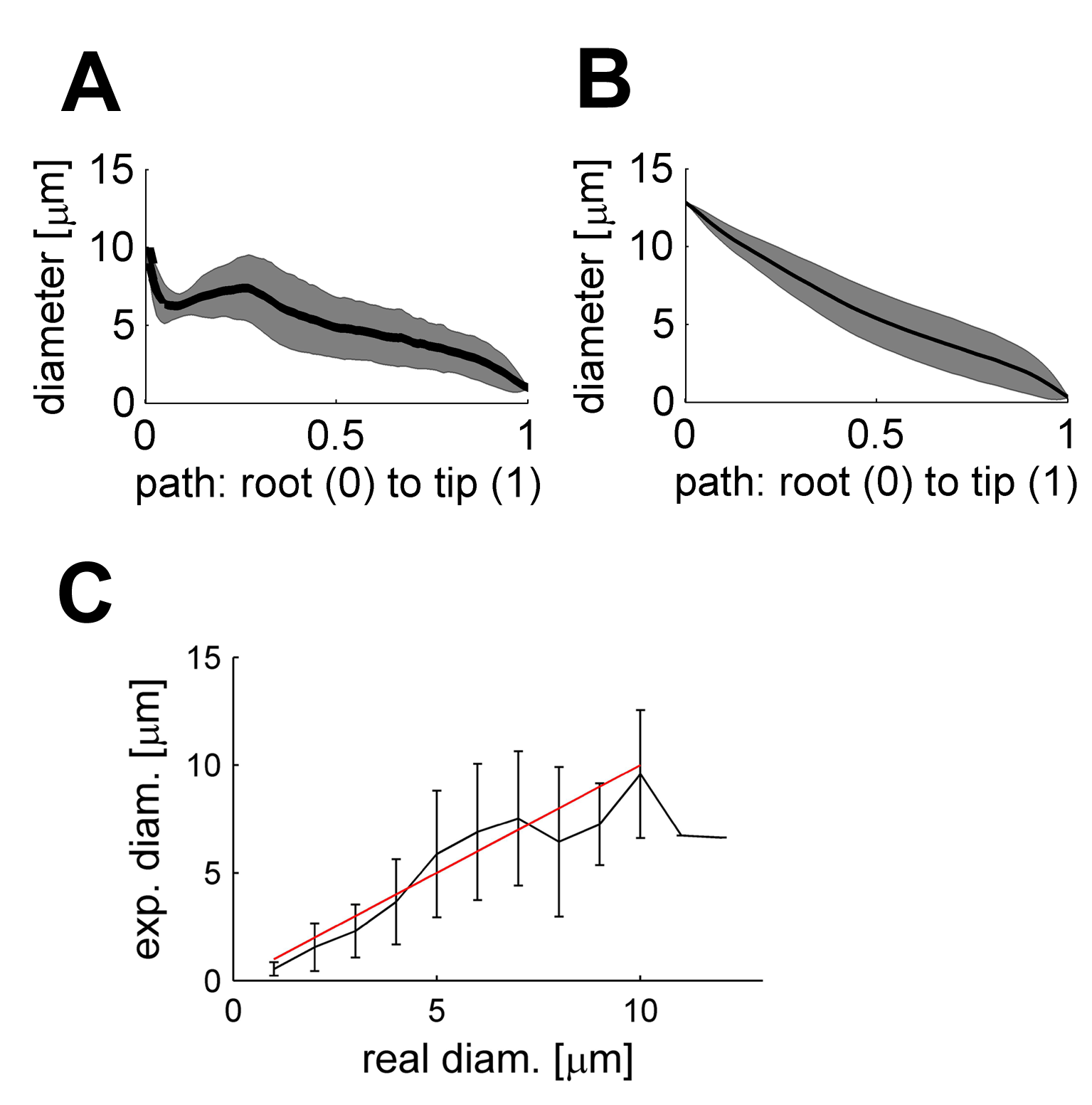


Figure S6
